# Simultaneous investigation of influenza and enteric viruses in the stools of adult patients consulting in general practice for acute diarrhea

**DOI:** 10.1186/1743-422X-9-116

**Published:** 2012-06-18

**Authors:** Christophe Arena, Jean Pierre Amoros, Véronique Vaillant, Katia Balay, Roxane Chikhi-Brachet, Laurent Varesi, Jean Arrighi, Thierry Blanchon, Fabrice Carrat, Thomas Hanslik, Alessandra Falchi

**Affiliations:** 1 INSERM, UMR-S 707, 75012, Paris, France; 2 UPMC Université Paris 06, UMR-S U707, 75012, Paris, France; 3 Laboratory of Virology, University of Corsica, Corsica, France; 4 Regional Observatory of Health of Corsica, Ajaccio, France; 5 Department of Infectious Diseases, Institut de Veille Sanitaire (InVS) (French Institute for Public Health Surveillance), Saint-Maurice, France; 6 National Reference Center for Enteric Viruses, Laboratory of Virology, CHU of Dijon, Dijon, France; 7 French National Agency for Research on AIDS and viral hepatitis, Paris, France; 8 Public health department, Saint-Antoine hospital, APHP, Paris, France; 9 UVSQ, Université de Versailles Saint Quentin, Versailles, France

**Keywords:** Influenza virus, Enteric virus, Stools, Co-infection, General practice

## Abstract

**Background:**

Gastrointestinal symptoms are not an uncommon manifestation of an influenza virus infection. In the present study, we aimed to investigate the presence of influenza viruses in the stools of adult patients consulting their general practitioner for uncomplicated acute diarrhea (AD) and the proportion of concurrent infections by enteric and influenza viruses.

**Method:**

A case-control study was conducted from December 2010 to April 2011. Stool specimens were collected and tested for influenza viruses A (seasonal A/H3N2 and pandemic A/H1N1) and B, and for four enteric viruses (astrovirus, group A rotavirus, human enteric adenovirus, norovirus of genogroups I – NoVGI - and genogroup II - NoVGII).

**Results:**

General practitioners enrolled 138 cases and 93 controls. Of the 138 stool specimens collected, 92 (66.7%) were positive for at least one of the four enteric viruses analysed and 10 (7.2%) tested positive for one influenza virus. None of these 10 influenza positive patients reported respiratory symptoms. In five influenza-positive patients (3.6%), we also detected one enteric virus, with 4 of them being positive for influenza B (2 had co-detection with NoVGI, 1 with NoVGII, and 1 with astrovirus). None of the 93 controls tested positive for one of the enteric and/or other influenza viruses we investigated.

**Conclusions:**

In this study we showed that the simultaneous detection of influenza and enteric viruses is not a rare event. We have also reported, for the first time in general practice, the presence of seasonal and pandemic influenza viruses in the stools of adult patients consulting for uncomplicated AD. A simultaneous investigation of enteric and influenza viruses in patients complaining of gastrointestinal symptoms could be useful for future studies to better identify the agents responsible for AD.

## Background

Gastrointestinal (GI) symptoms are not an uncommon manifestation of an influenza virus infection [1-3]. However, little is known about the GI pathogenesis of influenza viruses. It is possible that GI symptoms developed during the clinical course of influenza could either be a part of disease manifestation, due to the side effects of antibiotic treatment, or a co-infection with other diarrheal pathogens. Gastrointestinal manifestation associated with seasonal influenza has been recognised for more than 30 years [4]. During the influenza A epidemic of 1988 in Australia several children developed hemorrhagic gastritis of varying severity after a typical Influenza-like illness (ILI) [5]. Similarly, during the two epidemics in 1973 and 1974, influenza virus B was detected in hospitalised children who had abdominal pain, often severe enough to require differentiation from acute appendicitis, as a dominant symptom [1]. Less severe GI symptoms have been reported to occur in 20-30% of children with an influenza B infection [4,6,7]. Early epidemiologic study of the pandemic influenza A/H1N1 2009 virus suggested that it produced diarrhea, vomiting, or both, in ≈ 25% of case-patients [8].

However, fecal excretion of pandemic and seasonal influenza viruses has rarely been studied, and the lack of reports of co-infection among influenza and enteric viruses is probably because of reporting bias. Consequently it remains unknown whether co-infection with influenza pathogens in patients with GI symptoms represents rare events. Previous studies reported the detection of seasonal influenza in the stools of pediatric patients presenting concurrent acute diarrhea (AD) and ILI [9], and in the stools of hospitalised and outpatients presenting both GI and respiratory symptoms [10,11]. Influenza viral RNA was also detected in the stools of A/H1N1 2009 positive patients hospitalised due to the progression of acute gastroenteritis [12]. Previous studies showed that the avian influenza A/H5N1 virus can be detected in stools [13], and the presence of this virus was further demonstrated in the biopsy of the small and large intestines of fatal cases [14,15]. Other respiratory viruses have been found in stools, such as respiratory syncytial virus [16], SARS coronavirus [17], adenovirus [18] and bocavirus [19]. But, to our knowledge, there are no studies reporting the detection of Influenza viruses in the stools of adult patients consulting in general practice for acute diarrhea.

In the present study we aimed to investigate the presence of pandemic and seasonal influenza viruses in the stools of General Practitioners’ (GPs) adult patients presenting exclusively GI symptoms and the proportion of concurrent infections by enteric and influenza viruses by using a case control design.

## Results

### Samples collected

General practitioners enrolled 175 adult patients consulting for AD and 101 non diarrheal individuals, but we received stools samples from 138 cases and 93 controls. The two populations (cases and controls) presented similar demographic characteristics: median age of cases was 37 years [28 - 54] versus 39 years [29 - 54] for controls (p = 0.62); the proportion of women in the cases group was 45.9% versus 47.3% in the control group (p = 0.85).

### Virological findings

Of the 138 stool specimens collected, 92 (66.7%) were positive for at least one of the four enteric viruses analysed. Ten (7.2%) tested positive for one influenza virus, eight of them being positive for influenza virus B and two positive for influenza virus A (1 A/H1N12009 and 1 A/H3N2). Five influenza-positive patients (3.6%) showed a co-detection of one enteric virus (3 NoVGI, 1 NoVGII, and 1 astrovirus) (Table 1). None of the 93 controls were positive for either enteric and influenza viruses. Influenza viral concentration ranged from 2.5x10^4^ to 4.2x10^6^ PCR copies per gram of stool (Table 1).

**Table 1:** Demographical, clinical and virological data of the Influenza virus-positive patients

**Sex/Age**	**No of days from onset tostool collection**	**Mo of collection**	**Influenza Viral concentration (PCRcopies/g stool)**	**Underlying medical conditions**	**Gastrointestinal symptoms**	**Consistency of stool specimen**	**Duration of the enrolmentdiarrhea episode (days)**	**Duration of the enrolmentvomiting episode (days)**	**Duration of fever episode(days)**	**Supplementary medical examen**	**Influenza virus**	**Enteric virus**
M/24	2	January	2.8x10^4^	None reported	Abdominal pain, nausea, vomiting	Watery	3	3	6	None reported	A/H3N2	Negative
F/56	1	February	3.5x10^6^	Hypertension, osteoporosis, polymyalgia rheumatica	Abdominal pain, nausea	Watery	1	None reported	No fever	None reported	B	Negative
NA/>18	1	January	2.5x10^4^	None reported	Abdominal pain, nausea, vomiting	Loose	2	3	1	None reported	B	Norovirus GI
F/54	2	January	4.2x10^6^	None reported	Abdominal pain, nausea, vomiting	Watery	5	2	6	Fiberscope	B	Astrovirus
F/79	0	December	2.5x10^4^	Hypertension, osteoporosis	Abdominal pain, nausea, vomiting	Watery	1	1	1	None reported	B	Negative
M/22	7	January	3.2x10^4^	None reported	Abdominal pain, nausea	Watery	N/A	None reported	NA	None reported	B	Norovirus GI
F/19	3	February	4.0x10^4^	None reported	Abdominal pain, nausea	Watery	1	None reported	0	None reported	B	Negative
F/30	1	January	2.2x10^5^	Migraine	Abdominal pain, nausea	Watery	1	None reported	No fever	None reported	B	Negative
M/86	1	December	2.7x10^4^	None reported	Nausea	Watery	2	None reported	No fever	None reported	A/H1N1 2009	Norovirus GI
F/74	1	January	3.8x10^5^	Hypertension, osteoporosis, asthma	Abdominal pain, nausea, vomiting	Watery	2	1	1	None reported	B	Norovirus GII

### Characteristics of patients

In Table 2 we reported the median age, proportion of females, median duration of the enrolment diarrhea episode, proportion of patients presenting fever, and the median duration of fever after enrolment for 5 groups of patients: the ones who tested positive for both enteric and influenza viruses (n = 5), the ones who were positive only for influenza (n = 5), the sum of the two preceding groups, that is, the ones positive for the influenza virus with or without the co-detection of an enteric virus (n = 10), the ones who were only positive for at least one enteric virus (n = 87), and finally the ones who were negative for both enteric and influenza viruses (n = 41).

**Table 2:** Demographical and clinical data of patients

**Demographical and clinical data**	**Patients positive to influenza virus**			**Patients positive to enteric virus only**	**Patients negative to enteric and influenza viruses**
	**Patients positive to both influenza and enteric viruses**	**Patients positive to influenza virus only**	**Patients positive to at least one influenza virus**		
	**(n = 5)**	**(n = 5)**	**(n = 10)**	**(n = 87)**	**(n = 41)**
Median age (years) [IQ]*	64 [38 - 80]	30 [24 - 56]	43 [24 - 74]	35 [28 - 50]	44 [32 - 56]
**Females (%)**	2 (50.0%)	4 (80.0%)	6 (60.0%)	43 (49.4%)	18 (43.9%)
**Median duration of the enrolment diarrhea episode (days) [IQ]**	2 [2 - 3.5]	1 [1 - 1]	2 [1 - 2]	1 [1 - 2]	2 [1 - 4]
**Patients suffering of fever (%)**	4 (80.0%)	3 (60.0%)	7 (70.0%)	33 (37.9%)	9 (21.9%)
**Median duration of fever after enrollment (days) [IQ]**	1 [1 - 6]	1 [0 - 6]	1 [1 - 6]	1 [0 - 2]	1 [1 - 2]

* [IQ] = [Interquartile range].

Any significant differences, concerning demographical data (age and sex) have been pointed out between the five groups of patients described on Table 2. Concerning the clinical data reported in Table 2, patients with the detection of at least one enteric viruses (n = 87) seem to have a duration of fever after enrolment which is lower than patients who were positive for influenza, with or without co-detection of an enteric virus (n = 10) (OR = 0.49 [0.26-0.91]; p = 0.02). This significant difference holds true when we compare the same first group (n = 87) and the group of patients who tested positive for both enteric and influenza viruses (n = 5) (OR = 0.35 [0.13-0.91]; p = 0.03). Any significant differences for the duration of fever after enrolment have been highlighted between patients who tested positive for two enteric viruses (n = 11) with respect to patients who were positive for one enteric virus (n = 76) (p = 0.11).

None of the 10 influenza positive patients reported any respiratory symptoms.

Two influenza-positive patients declared the duration of enrolment fever episode of six days. The first one was a 24 year old man without underlying conditions and with the duration of enrolment diarrhea episode of 3 days, and who tested positive for the influenza virus A/H3N2 (viral concentration of 2.8x10^4^ PCR copies per gram of stool). The second one was a 54 year old woman without underlying conditions and with the duration of enrolment diarrhea episode of 5 days and who concomitantly tested positive for the influenza B and astrovirus (viral concentration of 4.2x10^6^ PCR copies per gram of stool).

## Discussion

The present study found evidence of the presence of seasonal and pandemic influenza viral RNA in 7.2% of adult patients (≥18 years old) consulting their GP for the typical and uncomplicated symptoms of AD during the ILI and AD outbreaks in France (http://www.sentiweb.fr). We have also reported the detection of enteric viruses in half of the patients who tested positive for influenza viruses. The most frequent combination was a co-detection with two agents, primarily influenza virus B plus NoVGI.

It is to be noted that in our study the most prevalent Influenza virus was influenza virus B, detected in 8 of 10 stool specimens positive for influenza viruses. These results seem to be in agreement with previous studies about the detection of influenza virus B in patients complaining of GI symptoms. The presence of influenza virus B in gastric mucosa has been previously reported among patients with GI symptoms without concurrent respiratory symptoms [20]. Similar results have been reported among hospitalised children infected with the influenza B virus for which abdominal pain was a dominant symptom, especially in older children [1]. As highlighted by Kaji et al. [21], GI symptoms were significantly more common in adult patients with a positive throat swab for the influenza B virus (GI = 23%), and with respect to the influenza A virus (GI = 6% for A/H3N2 and 4% for A/H1N1). Previously, the influenza B virus has been reported [9] in 81% (17/21) of influenza positive stools of pediatric patients (<6 years of age) with concurrent respiratory and GI symptoms. Interestingly, one of the influenza virus B strains detected among these pediatric patients was viable [9].

In this study we have also reported the detection of A/H3N2 and A/H1N1 2009 viral RNA in the stools of two patients with AD. The detection of the A/H3N2 virus in stool samples has been previously reported in six high-risk influenza adult patients [10] and in three young children [9] reporting ILI and diarrhea. Seasonal influenza viruses detection by RT-PCR in stools has also been reported in very young children presenting with ILI and AD between the ages of 5 weeks and 9 months [7]. Influenza virus A/H1N1 2009 was recovered from 16 (24.6%) stools of A/H1N1 2009 positive patients who were hospitalised due to the progression of acute gastroenteritis [12]. In another study, the authors showed a positive viral culture for A/H1N1 2009 in the stool of four patients presenting the highest viral load [22], suggesting the fecal shedding of viable pandemic viruses.

In this study, the overall proportion of co-detection of influenza and enteric viruses was 3.6%. We detected one enteric virus in 5/10 stool specimens of influenza-positive patients. Among them, four tested positive for the influenza B virus and one enteric virus (2 NoVGI, 1 NoVGII, and 1 astrovirus), and one for influenza A/H1N1 2009 (concomitantly with NoVGI). It is to be noted that although our sample was not large enough to make conclusions that are statistically approved, we can observe that patients who tested positive for both influenza and enteric virus were older (64 years [38-80]) than patients showing a single detection of influenza viruses (30 years [24-56]) and those ones positive for enteric viruses only (35 years [28-50]). To our knowledge, until now a co-detection of influenza viruses and enteric pathogens has rarely been reported. Co-infections between rotavirus and influenza viruses (6 influenza B and 1 influenza A) have been previously reported among 2.2% of hospitalised young children with gastroenteritis [23]. One case of co-infection with influenza A/H3N2 virus and norovirus has been reported in an elderly patient who developed diarrhea since day 3 and passed 3–4 episodes of watery/loose stool per day up to day 13 [24]. In the present study, the duration of fever seems to be shorter among patients who tested positive for at least one enteric virus with respect to patients positive for both enteric and influenza viruses. It is difficult to interpret this result given the low number of influenza-enteric co-detections and the low number of the ‘pure’ influenza-positive cases.

Finally, the explanation of the presence of seasonal (A and B) and pandemic A/H1N1 2009 influenza viruses RNA in the stools is not clear. As previously known, the avian influenza virus prefers to bind the α-2, 3-sialic acid receptor, while human Influenza viruses frequently bind the α-2, 6-sialic acid receptor. Recent evidence indicates that both types of receptors are expressed on the surfaces of *in vitro* differentiated intestinal epithelial cells [25-27], suggesting that both avian and human influenza viruses have the potential to infect and replicate in human intestinal epithelial cells. Recent data confirmed that human intestinal epithelial cells can be infected by the pandemic (H1N1) viruses and H9N2 viruses isolated from both humans and birds [28]. On the other hand, a recent study on adult hospitalised patients showed that a direct intestinal infection by seasonal influenza A viruses seems an unlikely explanation for the fecal detection of viral RNA in the patients reported [11]. Alternative explanations of influenza virus detection in stools could be the swallowing of virus-containing nasopharyngeal secretion or extrapulmonary virus dissemination via hematogeneous circulation.

This study has several limitations. First, the total proportion of viral co-detection was likely underestimated because we did not test other diarrheal pathogens. Thus, some cases of single infection in our study could be classified as multiple infections in studies which would include these other pathogens. Second, influenza virus cultures were not performed. However, to help us evaluate whether PCR signals were false positives, positive and negative controls were included in each PCR performed. The detection of influenza B has been performed by using two different primer pairs for the NS gene, and we detected influenza A by using two independent PCR assays for the detection of M gene and H gene. Third, respiratory samples were not collected. It is to be noted that the enrolment of patients was blind to any type of information related to respiratory tract infection, thus preventing potential bias.

## Conclusion

In conclusion, in this study we showed that the simultaneous detection of influenza and enteric viruses is not a rare event. We have also reported, for the first time in general practice, the presence of seasonal and pandemic influenza viruses in the stools of adult patients consulting for uncomplicated AD. This result could support the idea that the influenza virus could, on some occasions, be a responsible cause of gastroenteritis given the presence among some patients of diarrhea and the absence of any respiratory symptoms along with the absence of co-pathogens in 50% of them. More focused screening of fecal samples for the detection and isolation of influenza viruses in patients presenting with gastroenteritis will be required to demonstrate this additional potential disease association. The possible presence of infectious influenza viruses in fecal samples could create problems concerning infection control and highlights the importance of contact precaution when handling stools. Whereas influenza viruses are usually regarded to spread via direct contact with respiratory droplets, the possible fecal–oral transmission of influenza viruses has to be elucidated. This would have a number of implications for GP management of influenza virus infected patients, especially among patients at risk of severe influenza, in order to limit inadvertent human-to-human transmission. These cases are reported to highlight the potential clinical and infection control benefits of precisely knowing the true etiology of gastroenteritis-like symptoms. A simultaneous investigation of enteric and influenza viruses of patients complaining of GI symptoms could be useful for future studies in order to better identify the agents responsible for AD and to understand the potential mode of transmission and interaction of these viruses, especially during epidemic ILI and AD outbreaks.

## Methods

### Study design

A case–control study was conducted from December 2010 to April 2011. Sixty-three GPs from the French Sentinel Network [29] collected stools from adult patients (≥18 year old) consulting for AD (Sentinel network case-definition for AD: at least three daily watery or nearly so stools dating less than 14 days), and from controls. General practitioners had to enroll two patients per week, one case and one control. We excluded from the cases group: patients with inflammatory bowel disease, and patients with an obvious non-infectious etiology of diarrhea (recent use of antibiotics, colchicines, non-steroidal anti-inflammatory drugs, laxatives, recent administration of chemotherapy or radiotherapy). Controls were patients consulting their GP for non-GI diseases and not reporting GI symptoms during the month preceding the consultation. Data on the time of the onset of symptoms, reported symptoms, physical findings, gender, age, previous treatment, and medical attention before enrolment were collected by completing a case report form (CRF) for all participants who met the case definition. In addition, cases and controls sent a follow-up questionnaire the week after enrolment to indicate the duration of symptoms (for cases) and to ascertain whether an AD had occurred or not (for controls). The Hospital Ethic's Committee (CHU Saint-Antoine, Paris, France) approved the study. Oral consent was obtained from the patients at the time of inclusion, for their participation in the study and for the publication of the clinical and virological data.

### Sample analysis

Patients collected and sent, by postal mail, stool specimens in a triple packaging according to the instructions of the French National Reference Center for Enteric Viruses: the primary receptacle was a labeled primary watertight, leak-proof receptacle containing the specimen and without a transport medium. The receptacle was wrapped in absorbent material to absorb all fluid in case of breakage. A second durable, watertight, leak-proof receptacle was used to enclose and protect the primary receptacle. This secondary receptacle was placed in an outer shipping package bearing the United Nations packaging symbol (UN3373). In order to prevent the stool contamination by the patients’ respiratory secretions, general practitioners insisted on precautions when collecting the stool and this information was also indicated on the information letter we gave to patients.

All stool specimens were tested for influenza virus A (A/H1N1 2009 and A/H3N2) and Influenza B and for four enteric viral pathogens (astrovirus, group A rotavirus, human enteric adenovirus, and norovirus of genogroup I - NoVGI - and genogroup II - NoVGII).

Stool specimens were homogenised (20% wt/vol) in sterile water, centrifuged for 10 minutes at 3000 rpm and 200 μl of the clarified supernatants were subjected to nucleic acid extraction, using a QIAmp MinElute Virus Kit^®^ (QIAGEN, Courtaboeuf, France). Total nucleic acid was eluted in a final volume of 40 μl, of which 5 μl was used for PCR amplification. The efficiency of nucleic acid extraction was measured by real-time PCR amplification of the human GAPDH gene [30]. Influenza viruses A and B were detected by using two different real-time RT-PCRs [31]. Virus sub-typing (A/H1N1 2009 and A/H3N2) was performed by two real-time RT-PCRs [32,33]. Positive and negative controls were included in each RT-PCR. The copy number of influenza A and B viral RNA was determined against 10-fold serial dilution of external plasmid standards (from 2 x 10^8^ down to 2). The enteric viruses were detected by simultaneous amplification of nucleic acid through using the Seeplex® Diarrhea-V ACE assay (Seegene), and according to the manufacturer’s instructions. A recent study showed that the Seeplex® Diarrhea-V assay is sensitive, specific, convenient and reliable for the simultaneous detection of several viral pathogens found directly in stool specimens from patients with gastroenteritis [34].

### Statistical analysis

Continuous variables were described by median [interquartile range] and dichotomous data were described by proportions. Groups were compared by the Student test or Mann–Whitney test (as appropriate) for continuous variables. The Chi-2 or Fisher’s exact test (as appropriate) was used to compare dichotomous variables between groups, and the results are presented as Odds Ratios with their 95% confidence intervals (OR [95% IC]). All statistical analyses were two-tailed with a significance level (*P* value) of <0.05. Analyses were performed using STATA software (version 11.0, StataCorp LP, Texas, USA).

## Abbreviations

GI, GastroIntestinal; ILI, Influenza Like-Illness; AD, Acute Diarrhea; GP, General Practitioner; CRF, Case Report Form; NoVGI, NoroVirus of Genogroup I; NoVGII, NoroVirus of Genogroup II; GAPDH gene, GlycerAldehyde 3-Phosphate DeHydrogenase gene; OR, Odd Ratio; NS gene, Non Structural gene; M gene, Matrix gene; H gene, Hemagglutinin gene.

## Competing interests

The authors declare no conflict of interest.

## Author contributions

CA, JPA, VV, KB, RCB, LV, JA, TB, FC, TH, and AF co-conceived the study. CA and TH collected epidemiological and microbiological data. AF, LV and KB designed microbiology experiments. AF performed, analyzed and interpreted microbiology data. CA analyzed and interpreted epidemiological data. CA, JPA, VV, KB, RCB, LV, JA, TB, FC, TH, and AF contributed to writing the paper and approved the final manuscript.
